# Advanced Dental Materials: From Design to Application

**DOI:** 10.3390/ma17153667

**Published:** 2024-07-25

**Authors:** Josip Kranjcic, Tina Poklepovic Pericic

**Affiliations:** 1 Department of Prosthodontics, University Hospital Dubrava, Av. Gojka Šuška 6, 10000 Zagreb, Croatia; 2 Department of Fixed Prosthodontics, University of Zagreb School of Dental Medicine, Gunduliceva 5, 10000 Zagreb, Croatia; 3 School of Medicine, University of Split, Šoltanska 2, 21000 Split, Croatia

The title of this Special Issue is “Advanced Dental Materials: From Design to Application”. This is a very specific topic related to the rapid development of dentistry and, therefore, also of dental materials. The expectations of patients and the requirements of dentists are also increasing daily. Significant efforts are being invested in developing and improving the properties of the dental materials used in daily practice. The aesthetic properties of the materials are very important, but so are their mechanical and physical properties, i.e., their ability to withstand the stresses of a very dynamic environment—the oral cavity.

This Special Issue provides readers with up-to-date information on the properties of various materials: ceramics, acrylic resin, and composite materials, as well as dental alloys and their application in the field of prosthodontics using analog and digital technologies—additive and subtractive manufacturing technologies.

Twelve high-quality research papers were published in this Special Issue, covering a period of almost one and a half years. More than 60 authors from many institutions worldwide have contributed to the published articles.

Matta RE et al. (1) contributed a paper entitled “Stress Distribution within the Peri-Implant Bone for Different Implant Materials Obtained by Digital Image Correlation”; Bömicke W et al. (2) contributed a paper entitled “Bond Strength of Milled and Printed Zirconia to 10-Methacryloyloxydecyl Dihydrogen Phosphate (10-MDP) Resin Cement as a Function of Ceramic Conditioning, Disinfection and Ageing”; Berger L et al. (3) contributed a paper entitled “Effect of Luting Materials on the Accuracy of Fit of Zirconia Copings: A Non-Destructive Digital Analysis Method”; Pal A et al. (4) contributed a paper entitled “Fabrication of Ciprofloxacin-Immobilized Calcium Phosphate Particles for Dental Drug Delivery”; Vuksic J et al. (5,6) contributed papers entitled “The Influence of Contemporary Denture Base Fabrication Methods on Residual Monomer Content, Flexural Strength and Microhardness” and “Tensile Bond Strength between Different Denture Base Materials and Soft Denture Liners”; Zhang X et al. (7) contributed a paper entitled “Restorative Dental Resin Functionalized with Calcium Methacrylate with a Hydroxyapatite Remineralization Capacity”; Schröter FJ et al. (8) contributed a paper entitled “Pushout Bond Strength in Coronal Dentin: A Standardization Approach in Comparison to Shear Bond Strength”; Forysenkova AA et al. (9) contributed a paper entitled “Polyvinylpyrrolidone–Alginate–Carbonate Hydroxyapatite Porous Composites for Dental Applications”; Raszewski Z et al. (10) contributed a paper entitled “Bioactive Glass-Enhanced Resins: A New Denture Base Material”; Wakamori K et al. (11) contributed a paper entitled “Comparative Verification of the Accuracy of Implant Models Made of PLA, Resin, and Silicone”, and Nagata K et al. (12) contributed a paper entitled “Accuracy of Dental Models Fabricated Using Recycled Poly-Lactic Acid”.

This Special Issue provides readers with many scientific facts, but also serves as a link between science and clinical practice. We would also like to express our sincere thanks to all the authors who have contributed to this Special Issue. Their valuable research has made this Special Issue possible and we greatly appreciate their efforts.

